# Tuberculous Peritonitis

**Published:** 1908-11-07

**Authors:** 


### Surgery.

### TUBERCULOUS PERITONITIS.

This is a condition which presents itself in a great variety of forms—a fact which enables it to simulate more or less closely other abdominal lesions; and it is for this reason that, unless the surgeon is constantly on the watch for tuberculous peritonitis, he will assuredly meet with trouble.

Theoretically speaking, infection of the peritoneum with the tubercle bacillus may be direct or indirect—that is to say, it is possible to suppose that the infection is brought about either by bacilli residing in the vascular system or by the direct invasion of the serous covering by bacilli ingested into the intestine. On the whole, however, it is more likely that the latter is the real mode of infection, the bacilli leaving the intestine by the lymphatics and infecting the mesenteric glands in a manner analogous to that in which the bronchial glands are infected when the primary focus is in the respiratory tract. Infections of the peritoneum are more often direct than indirect. Appendicitis, for instance, is due to a direct invasion of the peritoneum covering this part of the intestine. But examples undoubtedly occur where the reverse is the case; thus, pneumococcal peritonitis must be regarded as a manifestation of a pneumococcal septicæmia, and, apart from perforating wounds, streptococcal peritonitis belongs essentially to the same category.

The onset of symptoms is nearly always insidious, the patient complaining of pain in the abdomen, of a more or less constant nature and usually not confined to any one particular part. There is always disturbance of the intestinal function, but this may be in the direction of constipation or of obstinate diarrhœa. In many cases as much may be learnt from the general appearance of the patient as from a study of the local condition. With few exceptions, it is a disease confined to young people; it is rare for it to occur in anyone over the age of forty. They are often of the delicate tuberculous type, with a flushed face.

The temperature chart does not differ from that obtained in other tuberculous affections. In a typical case there is a rise to about 101° in the evening, whereas the morning temperature may not be above the normal. A history of tuberculosis in the patient's family can often be obtained. If tubercle bacilli can be microscopically found in the fæces, the diagnosis is clinched.

Three clinical varieties of the disease are recognised according to the local condition found in the abdomen. They are (1) ascitic, (2) fibrous, and (3) caseous. In the first a large amount of serous fluid is secreted by the damaged peritoneum. The abdomen is then generally distended, and the condition has to be differentiated from an ascites due to cirrhosis of the liver. In each the abdomen is distended, and in each there is shifting dulness. When the patient is in the supine position the flanks are dull, but the front of the abdomen is tympanitic, owing to the fact that the coils of intestine float upon the fluid and lie in apposition to the posterior surface of the anterior abdominal wall. If the patient is turned on to his side, this state of affairs is reversed. But the differential diagnosis should not be difficult. Ascites occurs in elderly patients with an alcoholic history, in whom the liver may be felt to be enlarged, whereas tuberculous peritonitis attacks an entirely different class of individual.

Considerably greater difficulty is experienced if the effusion secreted by the peritoneum is encysted, especially if this occurs in the pelvis. In a young woman it is extremely difficult to differentiate it from an ovarian cyst. In both there is a cystic swelling in the pelvis; and, again, in both there may be amenorrhœa, a condition as constantly associated with tuberculosis generally as it is with ovarian cysts. Further, vaginal examination often gives no assistance. In fact, these two conditions may resemble one another so closely that it is often quite impossible to say which of the two is present without opening the abdomen. If the case be tuberculous there may be a coincident pleural effusion; and if this be withdrawn by paracentesis and examined microscopically a very large proportion of the cells in it will be found to be lymphocytes. This is highly suggestive of tuberculosis.

The fibrous variety is happily more rare; happily, because its results are more serious. In this, dense nodules are found, associated with fibrous adhesions, which bind down adjacent coils of intestine; so that often the first thing which calls the surgeon's attention to the condition is the onset of symptoms of intestinal obstruction.

The caseous variety is generally found in quite young children. In this, masses of soft caseating tuberculous material are deposited which tend to break down, and make their way to the surface at some part of the anterior abdominal wall, generally the umbilicus, leaving a sinus.

In either of the last two varieties the abdomen when examined will be found to have lost its natural elasticity. It may feel generally doughy, or distinct masses may be palpable. The omentum may be thickened and dependent (omental apron), or tucked up under the costal arch, where it forms a prominent elongated tumour (omental roll).

In recent years the treatment has consisted in opening the abdomen. Excellent results have followed this procedure in the ascitic cases. The exudate which has no bactericidal power is removed, and a fresh one is poured out, which is sufficiently powerful to overcome the toxins present. In many cases this treatment results in a permanent cure. But it must be freely confessed that no such happy ending can be looked for in the other varieties. Judging by the excellent results that are now being obtained from the administration of tuberculin in other surgical tuberculous conditions, such as tuberculous glands, it would seem worth while to give an extended trial to this method in tuberculous peritonitis.

